# Prevalence of Gastrointestinal Parasites in Pigs: A Preliminary Study in Tolon and Kumbungu Districts, Ghana

**DOI:** 10.1155/2023/1308329

**Published:** 2023-11-27

**Authors:** Francis Addy, Gideon Adu-Bonsu, Comfort A. Akurigo, Iddrisu Abukari, Hamidatu Suleman, Lydia Quaye

**Affiliations:** Department of Biotechnology and Molecular Biology, University for Development Studies, Tamale, Ghana

## Abstract

Pigs are important livestock that contribute to the economy and food security of Ghana, but the productivity of the sector is hindered by factors such as parasitic disease infections. Here, we detected the prevalence and polyparasitism of gastrointestinal parasites in pigs from selected farms in the Tolon and Kumbungu districts. Faeces of the animals were screened for parasite eggs using the sedimentation and flotation methods. From 56 pigs screened, 91.1% (CI = 95%; *χ*^2^ = 0.212) of them had parasitic infections, and affected pigs harboured 1–5 distinct parasites with the most occurring being *Strongyloides ransomi* (46.43%). Other parasites identified included *Hyostrongylusrubidus*, *Ascaris suum*, *Trichuris suis*, *Physocephalus sexalatus*, and Coccidia, among others. Parasitism was more frequent in pigs under 2 years (94%) than older ones (66.67%). The high prevalence of GI parasites was attributed to poor husbandry practices and inadequate of veterinary care for the animals. The situation can be improved by farmer education on good husbandry practices and regular deworming of pigs.

## 1. Introduction

Pigs are the most reared nonruminant (98.8%) animals in Ghana and continue to witness increasing production and consumption [1]. About 95% of the country's pork is produced by small scale and backyard pig farmers [2]. Pig farming contributes to Ghana's economy, and food and nutritional security of households. The pig industry has become a vital part of Ghana's agribusiness activities and a source of livelihood for several entrepreneurs who seek an alternative source of profitable business [2–4]. Pig production in Ghana is an old livestock enterprise, but it remains largely in the hands of rural farmers where animals are raised under poor husbandry systems.

The sector is challenged by many factors including a lack of improved breeding stock, unavailability of land and water, rapid urbanization, piglet mortality, high feed cost, and disease infections [4–6]. Among infectious disease agents in livestock, gastrointestinal parasites are noted to be very devastating and could cause severe loss to the industry [7] Especially in the tropics where high humidity and temperature conditions support the spread of parasitic infections [8], a poor husbandry system such as in sub-Saharan Africa may even compound the impact on production animals [9–12]. Gastrointestinal parasites, i.e., helminths and protozoans, tend to hinder profitable pig production; causing poor feed conversion [10], delayed estrus, and conception rate [13], producing lesions that may lead to condemnation of organs and or carcasses [14] and even death [15]. Also, pigs are host to a number of zoonotic parasites that have an impact on public health [16–18]. Meanwhile, the epidemiology of intestinal infections in pigs is not well understood in many localities, but this is essential if good disease management strategies are to be developed. For instance, in Ghana where pig production is done largely on a subsistence scale in rural settings, it will be useful to build a comprehensive database of the disease situation and dynamics in the various ecological zones where pigs are raised.

The present work was carried out as a preliminary to look at the prevalence, intensity, and species of intestinal parasites in circulation in pigs raised in rural-semi-intensive systems in two administrative districts of northern Ghana. The outcome points to a high parasitic infection in pigs which could severely impact the productivity of the animals.

## 2. Materials and Methods

### 2.1. Study Area

The research was conducted in the Tolon and Kumbungu districts of Ghana. The two districts are jointly characterized by Guinea Savanna woodland interspersed with short drought-resistant trees and grassland. The area experiences a unimodal rainfall with a mean annual range of 1000 mm–1200 mm [19, 20]. According to the 2021 Population and Housing Census, the Tolon and Kumbungu districts, respectively, have a human population of 118,101 and 110,586, and 641 and 191 pig population [19–21].

### 2.2. Study Design and Sample Collection

The study was conducted in March 2022 at eight (8) piggeries in the two districts using the snowball sampling technique. This sampling technique was employed due to the absence of pig farmer's registry with the appropriate authorities.

Faecal samples were taken from each pig at each piggery except for pregnant sows, and piglets that were younger than 2 months. Fresh faeces were taken from the ground (without debris) immediately after defecation and put in a sterile container. Faecal samples were stored and maintained in cold boxes until they were processed.

In all the pig farms studied, farmers confirmed that no anthelmintic had been administered within the past 90 days to the study. Information such as sex, age, deworming status, frequency of deworming, and type of anthelmintics used were enquired of farmers and recorded accordingly.

### 2.3. Identification of Worm Species

Sedimentation and centrifugal flotation methods were employed in the identification of parasites in all faecal samples as described by Tagesu [22] with some modifications.

#### 2.3.1. Sedimentation Method

With the sedimentation method, 1 g of faeces was homogenized in 10 ml of distilled water. The suspension was filtered through a kitchen strainer into a sterile container and allowed to sediment for 5 min. The sediment was resuspended in 3 ml of dH_2_O and allowed to sediment for another 5 min, and the supernatant was discarded. The sediment was then stained with 1% (w/v) methylene blue. A drop (30–50 *μ*l) of the stained sediment was transferred to a microscope slide using a pipette and covered with a cover slide for microscopical screening.

#### 2.3.2. Centrifugal Flotation Method

In this method, 200 mg of faecal matter was homogenized in 3 ml of dH_2_O and centrifuged at 629 g for 7 min, and the supernatant was decanted. Afterwards, 3 ml of saturated NaCl (flotation solution) was added, shaken vigorously, and filled to the brim to form a meniscus. A coverslip was placed on top, and the mixture was allowed to stand for 10 min. The coverslip was mounted on a microscope for egg identification. Eggs were detected and species determined at 10x and 40x magnification, respectively, guided by Thienpont et al. [23].

### 2.4. Data Management and Statistical Analyses

Demographics of animals and their infection status were entered into Microsoft Excel LTLC Professional Plus 2021 and were also used in the tabulation and representation of results in charts and graphs. IBM SPSS Statistics V20.0 (IBM Corporations, New York, USA) was used for all other statistical inferences. The association between the risk factors and the outcome variables was assessed using the chi-square (*x*^2^) test. For all analyses, a *p* value < 0.05 was considered as significant. An animal was flagged positive if at least one parasite was identified in its faeces, and prevalence was calculated as the percentage of the number of animals infected per number of animals screened.

## 3. Results

### 3.1. Demographics of Study Subjects

From the eight piggeries studied, 56 pigs were screened of which 37 (66.07%) were sows and 19 were (33.93%) boars, between the ages of 3 months and 4 years. The modal and mean ages of the pigs were 6 months and 9 months old, respectively.

### 3.2. Incidence of Parasite Infestation

**Figure 1:**
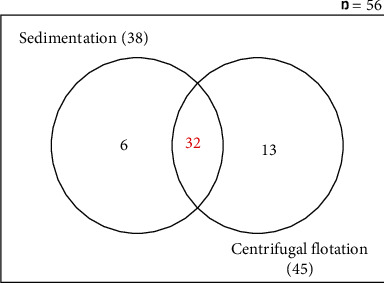
Detection of eggs of parasitic worms in pig faeces as determined by sedimentation and flotation screening methods.

The sedimentation method showed a prevalence of 67.86% (38/56) of parasite infestation whereas the flotation method revealed 80.36% (45/56) prevalence. Generally, parasitic infection in the pigs was very common as 51/56 swine, representing 91.07% (CI = 95%; *χ*^2^ = 0.212), were infected (see Figure 1).

Male pigs recorded relatively higher parasitic prevalence, 94.74% (18/19), than that in females, 89.19% (33/37) at *x*^2^ = 0.475 and OR = 2.182 (see Table 1). Infection was more common in pigs aged ≤ 2 years than older ones (94.00 vs. 66.67% (Table 1).

### 3.3. Identified Parasites and Occurrence of Polyparasitism

**Figure 2:**
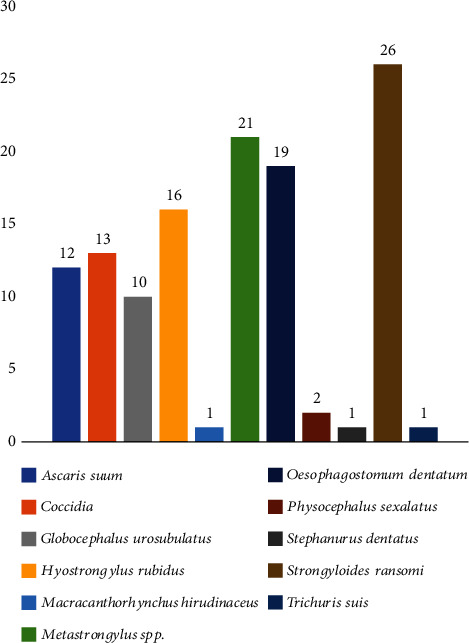
Parasites identified in screened faeces and their occurrence in pigs in the study area.

**Figure 3:**
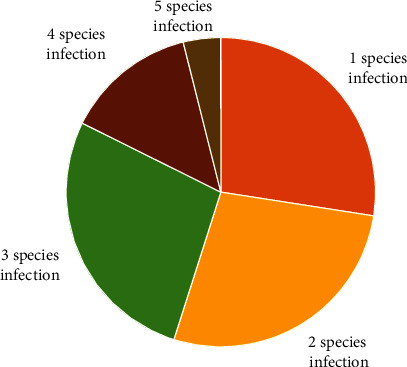
Mono- and- polyparasitism of worms in pigs.

Across the infected animals, 10 distinct parasitic worm species and a protozoan were identified (Figure 2). These include nine families of nematodes, an acanthocephalan, *Macracanthorhynchus hirudinaceus*, and the protozoan Coccidia. The most occurring parasite was *Strongyloides ransomi* (46.43%) whereas *Macracanthorhynchus hirudinaceus*, *Stephanurus dentatus*, and *Trichuris suis* (1.79%) were the least. The infected pigs frequently suffered polyparasitism (infection by two or more parasite species); up to 72.55% harboured 2-5 different parasitic worm species, whereas 27.45% harboured single parasitic worm species (see Figure 3).

Because of the advantages and limitations of each method of identification, they may not all be successful in identifying specific parasitic ova [24, 25].  Table 2 provides information on the screening method that identified individual parasites from the present study.

## 4. Discussion

Parasitic infection in rural pigs is an important feature, especially, in resources poor settings, where it has been shown to greatly affect productivity of the animals [7]. The prevalence of parasitic infections recorded in the present study is not different from that reported by Permin et al. two decades ago from the Upper East Region [26], 91.07% vs. 91.00%, all in northern Ghana (Guinea savannah ecological zone). While our account is a preliminary investigation and restricted to a small area and animal numbers, it is still intriguing to record such a high occurrence intensity of parasitic helminths in pigs in this half of Ghana despite the long-term knowledge of their abundance in the area. The observed persistence of the parasites was attributed to poor pig husbandry which leads to poor hygiene of their food and water and contamination of soil [27, 28] and poor knowledge by farmers on the involvement of pigs in parasite transmission. In another cross-sectional surveillance in the forest zone, Atawalna et al. [29] reported 28% parasitic infection prevalence in pigs in the Ejisu municipality of the Ashanti Region, Ghana. Within the subregion, high prevalence has been equally reported in Cameroon (74.7%), Nigeria (71.9–80%), South Africa (79.2%), Ethiopia (61.8%), and Rwanda (84.6%) [10, 12, 30–33]. Although coprology, as used in the present study, is a useful tool in detecting and quantifying endoparasites, it has its own limitations of being unable to detect all parasites, e.g., lung and stomach nematodes as observed by Gassó et al. [34].

Age-group and sex-specific prevalence were not statistically significant (*p* > 0.05) in the present study. The relative higher prevalence in young pigs may be attributed to susceptible or compromising immune system. Although comparatively lower (per the dictate of the present study), 66.67% prevalence in pigs that were more than 24-month-old can be attributed to older animals picking up more infection as a result of the intensity and length of feeding habits as opined by Nwokoye et al. [12], but a larger sample size and wet season study will be needed to validate our observation.

In the present study, 11 distinct parasites were identified from screened faecal samples including the zoonotic *Ascaris suum*, *Oesophagostomum dentatum*, *Strongyloides ransomi*, *Macracanthorhynchus hirudinaceus*, and *Trichuris suis*. The presence of these zoonotic helminths has been reported in earlier studies in Upper East [26] and Ejisu [29] in Ghana as well as other countries in the subregion including Cameroon and Nigeria. The transmission of these parasites across hosts is sustained by poor environmental hygiene like open defecation [35], a practice that is not uncommon in the Northern Region of Ghana [36–38]. The transmission of these zoonotic parasites may get complex if not curbed as, for instance, an aberrant *A. suum* infection has been reported in a dog in China [39] underpinning the domestic animal as a potential host alongside rodent reservoirs [13]. The other identified parasites, which have also been identified within Ghana and outside by other researchers, are equally important as they cause significant losses in pigs [16, 28, 40] although farmers may be unaware due to subclinical infections [40]. The impact of polyparasitism on the affected pigs was not examined in the present study, but it is thought to be significant due to the higher rate polyparasitism seen. As demonstrated by Serrano and Millán [41], multiple pathogen infections are the usual occurrence, and where a host is affected by high diversity of pathogen community, they present prominent impacts on host animal health. Among the infected pigs, 72.55% had coinfection, thus, infected by two or more parasite species. The rate of mixed parasitosis infection was higher than the 7% reported by Nwokoye et al. [12] in Ghana and 31.7% in Nigeria [12] but less than the 84.1% mixed infection reported in Rwanda [33].

The socioeconomic, cultural and moral roles of humans are equally at play in the transmission of parasites in the study area, and for this, all stakeholders are needed on board to curb this increasing menace of parasitosis by primarily educating pig farmers on environmental hygiene and need for veterinary services.

## 5. Conclusions

Although preliminary, the study revealed high gastrointestinal parasite prevalence in pigs in Tolon and Kumbungu districts of northern Ghana. Among the eleven distinct parasites identified are *A. suum* and *T. suis* which have zoonotic potentials. However, all swine parasites are of great public health importance, and for that reason, adequate efforts must be made to reduce to the barest minimum or eradicate transmission outright.

## Figures and Tables

**Table 1:** Infection status and risk estimate of pig to infection by sex and age.

Animal (ŋ)	Animals with parasitic worm eggs (%)	*x* ^2^	OR/risk estimate
*Sex*			
Female (37)	33 (89.19%)	0.475	2.182
Male (19)	18 (94.73%)		
Total (56)	51 (91.07%)		
*Age group*			
<1 year (31)	29 (93.55%)	0.085	n/d
1–2 years (19)	18 (94.74%)		
>2 years (6)	4 (66.67%)		
Total (56)	51 (91.07%)		

Confidence interval = 95%; OR = odds ratio; *x*^2^ = Pearson's chi-square; n/d = not determined.

**Table 2:** Method of screening that identified the various gastrointestinal parasites.

S/N	Parasite identified	Phylum	Method of identification	
			Sedimentation	Centrifugal flotation
1	*Ascaris suum*	Nematoda	×	✓
2	Coccidia	Apicomplexa	✓	×
3	*Globocephalus urosubulatus*	Nematoda	✓	✓
4	*Hyostrongylus rubidus*	Nematoda	✓	✓
5	*Macracanthorhynchus hirudinaceus*	Acanthocephala	✓	×
6	*Metastrongylus* spp.	Nematoda	✓	✓
7	*Oesophagostomum dentatum*	Nematoda	✓	✓
8	*Physocephalus sexalatus*	Nematoda	✓	×
9	*Stephanurus dentatus*	Nematoda	×	✓
10	*Strongyloides ransomi*	Nematoda	✓	✓
11	*Trichuris suis*	Nematoda	✓	×

✓ = screening method that identified the said parasite; × = not identified.

## Data Availability

Data used to support the findings of this study are available upon request. Contact Francis Addy, PhD (faddy@uds.edu.gh).

## Additional Points

*Recommendations*. (i) Future studies can be done on larger sample sizes in a retrospective study. (ii) The current study was conducted in the dry season, and another study can be done in the wet season to compare and contrast the findings and also make statistical inferences of the numbers. (iii) Future works can use molecular methods to identify parasite species precisely and study their inter and intra genetic variabilities.

## References

[1] Ghana Statistical Service (2020). *Ghana Census of Agriculture*.

[2] Boateng M., Amoah K. O., Atuahene P. Y., Okai D. B., Achemapong M. (2021). Assessment of the status of pig production in the Greater Accra region of Ghana. *Ghanaian Journal of Animal Science*.

[3] Aryee S. N. D., Osei-Amponsah R., Adjei O. D., Ahunu B. K., Skinner B. M., Sargent C. A. (2019). Production practices of local pig farmers in Ghana. *International Journal of Livestock Production*.

[4] Banson K. E., Nguyen N., Sun D. (2018). Strategic management for systems archetypes in the piggery industry of Ghana—a systems thinking perspective. *Systems*.

[5] Sekyere J. O., Adu F. (2015). Prevalence of Multidrug Resistance among *Salmonella enterica* Serovar Typhimurium Isolated from Pig Faeces in Ashanti Region, Ghana. *Cibtech Journal of Zoology*.

[6] Adzitey F. (2013). Animal and meat production in Ghana-an overview. *Journal of World's Poultry Research*.

[7] Yadav S., Gupta A., Choudhary P., Kumar P. (2021). Prevalence of gastrointestinal helminths and assessment of associated risk factors in pigs from Rajasthan districts, India. *Journal of Entomology and Zoology Studies*.

[8] Taylor M. A., Coop R. L., Wall R. L. (2015). *Veterinary Parasitology*.

[9] Swai E. S., Rukambile E. J., Chengula A. A., Wilson T. R. (2017). Endo-, ecto- and haemo-parasites of pigs in Tanzania. *International Biology Review*.

[10] Abonyi F. O., Njoga E. O. (2020). Prevalence and determinants of gastrointestinal parasite infection in intensively managed pigs in Nsukka agricultural zone, southeast, Nigeria. *Journal of Parasitic Diseases*.

[11] Pinilla J. C., Morales E., Muñoz A. A. F. (2021). A survey for potentially zoonotic parasites in backyard pigs in the Bucaramanga metropolitan area, Northeast Colombia. *Vet World*.

[12] Nwokoye C. I., Onusiriuka B., Yahaya U., Dikwa K. B. (2021). A study of intestinal helminths of swine from Chikun and Jema’a local government areas of Kaduna State, Nigeria. *FUDMA Journal of Sciences*.

[13] The Center for Food Security & Public Health (2021). *Zoonotic Diseases of Swine*.

[14] Adhikari R. B., Adhikari Dhakal M., Thapa S., Ghimire T. R. (2021). Gastrointestinal parasites of indigenous pigs (Sus domesticus) in south-central Nepal. *Veterinary Medicine and Science*.

[15] Sowemimo A. O., Asaolu S. O., Adegoke F. O., Ayanniyi O. O. (2012). Epidemiological survey of gastrointestinal parasites of pigs in Ibadan, Southwest Nigeria. *Journal of Public Health and Epidemiology*.

[16] Kouam M. K., Ngueguim F. D. (2022). Prevalence, intensity, and risk factors for helminth infections in pigs in Menoua, Western Highlands of Cameroon, with some data on protozoa. *Journal of Parasitology Research*.

[17] Dey T. R., Dey A. R., Begum N., Akther S., Barmon B. C. (2014). Prevalence of end parasites of pig at Mymensingh, Bangladesh. *IOSR Journal of Agriculture and Veterinary Science*.

[18] Ayinmode A. B., Obebe O. O., Olayemi E. (2017). Prevalence of potentially zoonotic gastrointestinal parasites in canine faeces in Ibadan, Nigeria. *Ghana Medical Journal*.

[19] Ghana Statistical Service (GSS) District Analytical report of Tolon district. https://www2.statsghana.gov.gh/docfiles/2010_District_Report/Northern/TOLON.pdf.

[20] Ghana Statisical Service (GSS) (2014). *District Analytical report of Kumbungu district*.

[21] Ghana Statistical Service (GSS) (2021). *Population of Regions and Districts*.

[22] Tagesu A. (2018). Manual guidance of veterinary clinical practice and laboratory. *International Journal of Veterinary Sciences Research*.

[23] Thienpont D., Rochette F., Vanparijis O. F. J. (2003). Diagnosing Helminthiasis by Coprological Examination. *Diagnosing Helminthiasis by Coprological Examination*.

[24] Dryden M. W., Payne A. P. (2014). *Faecal examination techniques*.

[25] Abede W., Esayas G. (2001). *Survey of ovine and caprine gastrointestinal helminthosis in eastern part of Ethiopia during the dry season of the year*.

[26] Permin A., Yelifari L., Bloch P., Steenhard N., Hansen N. P., Nansen P. (1999). Parasites in cross-bred pigs in the Upper East Region of Ghana. *Veterinary Parasitology*.

[27] Levy K., Hubbard A. E., Nelson K. L., Eisenberg J. N. S. (2009). Drivers of water quality variability in northern coastal Ecuador. *Environmental Science & Technology*.

[28] Larbi J. A., Addo S. O., Ofosu-Amoako G., Offong U. C., Odurah E. M., Akompong S. K. (2022). Burdens of Ascaris spp. and Cryptosporidium spp. parasites in farm pigs in Ghana. *Veterinary Medicine and Science*.

[29] Atawalna J., Attoh-Kotoku V., Folitse R. D., Amenakpor C. (2016). Prevalence of gastrointestinal parasites among pigs in the Ejisu municipality of Ghana. *Scholars Journal of Agriculture and Veterinary Sciences*.

[30] Kouam M. K., Ngueguim F. D., Kantzoura V. (2018). Internal parasites of pigs and worm control practices in Bamboutos, Western Highlands of Cameroon. *Journal of Parasitology Research*.

[31] Nwafor I. C., Roberts H., Fourie P. (2019). Prevalence of gastrointestinal helminths and parasites in smallholder pigs reared in the central Free State Province. *Onderstepoort Journal of Veterinary Research*.

[32] Geresu M. A. (2015). Prevalence and associated risk factors of major gastrointestinal parasites of pig slaughtered at Addis Ababa Abattoirs Enterprise, Ethiopia. *Journal of Veterinary Science and Technology*.

[33] Tumusiime M., Ntampaka P., Niragire F., Sindikubwabo T., Habineza F. (2020). Prevalence of swine gastrointestinal parasites in Nyagatare district, Rwanda. *Journal of Parasitology Research*.

[34] Gassó D., Feliu C., Ferrer D. (2015). Uses and limitations of faecal egg count for assessing worm burden in wild boars. *Veterinary Parasitology*.

[35] Nejsum P., Betson M., Bendall R. P., Thamsborg S. M., Stothard J. R. (2012). Assessing the zoonotic potential of Ascaris suum and Trichuris suis: looking to the future from an analysis of the past. *Journal of Helminthology*.

[36] Trimmer J. T., Kisiangani J., Peletz R. (2022). The impact of pro-poor sanitation subsidies in open defecation-free communities: a randomized, controlled trial in rural Ghana. *Environmental Health Perspectives*.

[37] United State Agency for International Development (USAID) (2021). *The Challenges of Sustaining Open Defecation Free (ODF) Communities in Rural Ghana*.

[38] Adzawla W., Alhassan H., Jongare A. I. (2020). Explaining the effects of socioeconomic and housing characteristics on the choice of toilet facilities among Ghanaian households. *Journal of Environmental and Public Health*.

[39] Xie Y., Liu Y., Gu X. (2020). First report on aberrant Ascaris suum infection in a dog, China. *Parasites & Vectors*.

[40] Karang A. K., Karang K., Swacita I. B. N. (2017). Reducing zoonotic and internal parasite burdens in pigs using a pig confinement system. *Vet World*.

[41] Serrano E., Millán J. (2014). What is the price of neglecting parasite groups when assessing the cost of co-infection?. *Epidemiology and Infection*.

